# Screening and identification of novel B cell epitopes of *Toxoplasma gondii* SAG1

**DOI:** 10.1186/1756-3305-6-125

**Published:** 2013-04-30

**Authors:** Yanhua Wang, Guangxiang Wang, Delin Zhang, Hong Yin, Meng Wang

**Affiliations:** 1 State Key Laboratory of Veterinary Etiological Biology, Lanzhou, 730046, China; 2 Key Laboratory of Veterinary Public Health of the Ministry of Agriculture, Lanzhou, 730046, China; 3 Lanzhou Veterinary Research Institute, Chinese Academy of Agricultural Sciences, Lanzhou, 730046, China

**Keywords:** *Toxoplasma gondii*, SAG1, Epitope, Pig antibodies

## Abstract

**Background:**

The identification of protein epitopes is useful for diagnostic purposes and for the development of peptide vaccines. In this study, the epitopes of *Toxoplasma gondii* SAG1 were identified using synthetic peptide techniques with the aid of bioinformatics.

**Findings:**

Eleven peptides derived from *T. gondii* SAG1 were assessed by ELISA using pig sera from different time points after infection. Four (PS4, PS6, PS10 and PS11), out of the eleven peptides tested were recognized by all sera. Then, shorter peptides that were derived from PS4, PS6, PS10 and PS11 were predicted using bioinformatics and tested by experimentation. Four out of nine shorter peptides were identified successfully (amino acids 106–120, 166–180, 289–300 and 313–332).

**Conclusions:**

We have precisely located the epitopes of *T. gondii* SAG1 using pig sera collected at different time points after infection. The identified epitopes may be useful for the further study of epitope-based vaccines and diagnostic reagents.

## Findings

*Toxoplasma gondii* is an obligate intracellular parasite that infects a variety of mammals and birds, causing toxoplasmosis [1,2]. *T. gondii* is an important food-borne parasite, and the primary route of transmission from animals to humans is through the consumption of infected meat [3,4]. In some countries, pork is the most common meat consumed, and some ethnic groups consume raw pork; thus, pigs are considered to be the primary source of human infection with *T. gondii*[5]. In addition, toxoplasmosis is a source of significant economic losses for swine farmers [6]. Therefore, the development of effective diagnostic reagents or vaccines for controlling this infection is required. Attempts to develop a peptide-based vaccine for *T. gondii* have focused on SAG1 and shown encouraging results [7]. Furthermore, the multiepitope antigen is one of the most promising antigens for the serodiagnosis of toxoplasmosis. Thus, it is very important to determine the precise sequences against which effective immune responses are directed. SAG1 epitopes have been studied by different research groups [8-10]. However, it is still unclear which SAG1 peptides are recognized by antibodies from pigs infected with *T. gondii.* Therefore, B cell epitopes of SAG1 were analyzed using a synthetic peptide technique in combination with software-based prediction.

### Serum samples

A total of 51 *T. gondii*-positive sera, which had been previously collected from experimentally infected pigs by our lab, were investigated. Twelve pig serum samples were collected at the time of presentation of clinical symptoms (G1); 18 follow-up specimens were taken on days 14 to 35 after the onset of symptoms (G2), and 21 further serum samples were taken on days 60 to 120 after the onset of symptoms (G3). *Toxoplasma* IgM and IgG antibodies were confirmed by *T. gondii* lysate antigen-ELISA. The serum samples in G1 and G2 were positive for IgM and IgG against *T. gondii*. The serum samples in G3 were only positive for IgG against *T. gondii*. Ten sera that were negative for *T. gondii* IgM and IgG were used as controls. The experimental protocol was approved by the Ethical Committee of the Lanzhou Veterinary Research Institute, Chinese Academy of Agricultural Sciences.

### Synthetic peptides

Based on the sequence of *T. gondii* SAG1 (Genbank Accession No. FJ455529), 20 non-overlapping or overlapping 12–36 mer peptides were synthesized by GL Biochem Ltd (Shanghai, China). Peptide sequences are shown in Table 1.

**Table 1:** Sequences of synthesized peptides

**Peptides**	**Start and end position**	**Sequence**
PS1	1–30 aa	MSVSLHHFIISSGFLASMFPKAVRRAVTAG
PS2	31–60 aa	VFAAPTLMSFLRCGAMASDPPLVANQVVTC
PS3	61–90 aa	PDKKSTAAVILTPTENHFTLKCPKTALTEP
PS4	91–120 aa	PTLAYSPNRQICPAGTTSSCTSKAVTLSSL
PS5	121–150 aa	IPEAEDSWWTGDSASLDTAGIKLTVPIEKF
PS6	151–180 aa	PVTTQTFVVGCIKGDDAQSCMVTVTVQARA
PS7	181–210 aa	SSVVNNVARCSYGANSTLGPVKLSAEGPTT
PS8	211–240 aa	MTLVCGKDGVKVPQDNNQYCSGTTLTGCNE
PS9	241–270 aa	KSFKDILPKLSENPWQGNASSDNGATLTIN
PS10	271–300 aa	KEAFPAESKSVIIGCTGGSPEKHHCTVQLE
PS11	301–336 aa	FAGAAGSAKSSAGTASHVSIFAMVTGLIGSIAACVA
PS4-1	91–105 aa	PTLAYSPNRQICPAG
PS4-2	106–120 aa	TTSSCTSKAVTLSSL
PS4-3	100–114 aa	QICPAGTTSSCTSKA
PS6-1	151–165 aa	PVTTQTFVVGCIKGD
PS6-2	159–173 aa	VGCIKGDDAQSCMVT
PS6-3	166–180 aa	DAQSCMVTVTVQARA
PS10-1	271–305 aa	KEAFPAESKSVIIGC
PS10-2	281–294 aa	VIIGCTGGSPEKHH
PS10-3	289–300 aa	SPEKHHCTVQLE
PS11-1	301–315 aa	FAGAAGSAKSSAGTA
PS11-2	313–332 aa	GTASHVSIFAMVTGLIGSIA
PS11-3	306–320 aa	GSAKSSAGTASHVSI

### ELISA analysis

**Figure 1:**
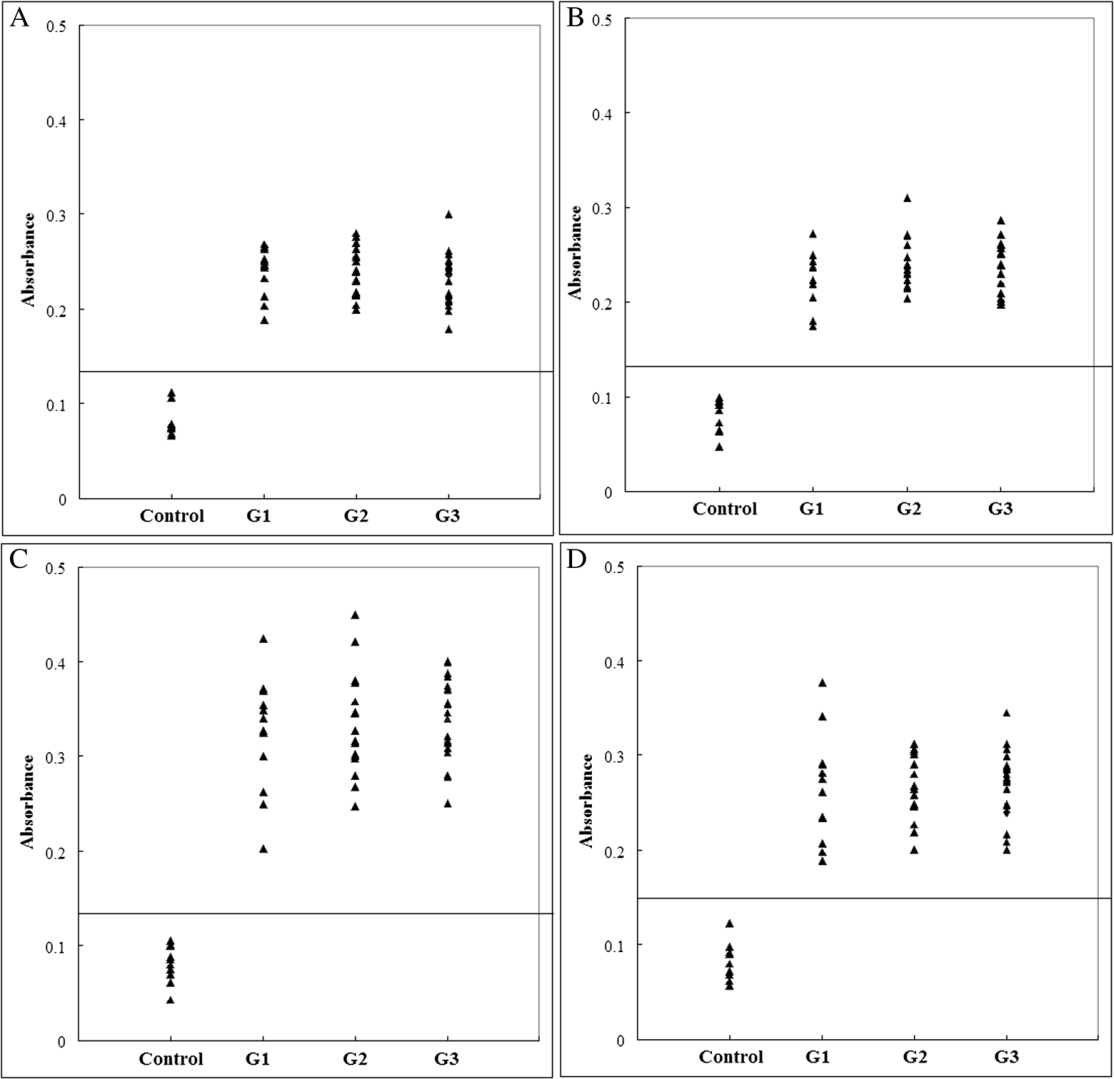
**ELISA of IgG antibodies against different peptides in four groups of pig sera.** (**A**), (**B**), (**C**) and (**D**) show the absorbances targeting to PS4, PS6, PS10 and PS11, respectively. The cut-off point for the assay is indicated by the horizontal line.

ELISA for each peptide were performed as described by Cardona *et al*. [10], except that microplates were coated with 10 μg/ml peptide and peroxidase-conjugated anti-pig IgG (1:4000) was used as the secondary antibody. PS1 was negative to all sera; PS2, PS3, PS5, PS7, PS8 and PS9 were recognized by few or partial sera (PS2: 5/51, PS3: 13/51, PS5: 24/51, PS7: 32/51, PS8: 6/51 and PS9: 25/51), and only Four peptides (PS4, PS6, PS10 and PS11) were recognized by all sera (Figure 1). For each of the 4 peptides, no significant differences were observed between the mean absorbances of the three groups, as determined by an ANOVA statistical test. The mean absorbance ± standard deviation of one peptide (amino acids 271–300) was significantly higher (0.337 ± 0.053) than that of the other peptides (PS4: 0.235 ± 0.025, p = 0.000; PS6: 0.237 ± 0.0028, p = 0.000; PS11: 0.266 ± 0.039, p = 0.001). Five peptides (amino acids 61–80, 181–200, 241–260 and 301–320) derived from SAG1 were determined to be B cell epitopes, and one peptide (amino acids 301–320) was the peptide that was most strongly recognized by sera from patients with human ocular toxoplasmosis [10]. A robust immunological response to the SAG1 is associated with chronic *Toxoplasma* infection in humans [11]. However, we found that peptides derived from SAG1 were capable of being recognized by pig sera from different time points after infection, which is different from previous reports. The discrepancy could be explained by differences in parasite strains, by using different animal models as well as by the different MHC-types between human and pig.

### Precise definition of the epitopes

**Figure 2:**
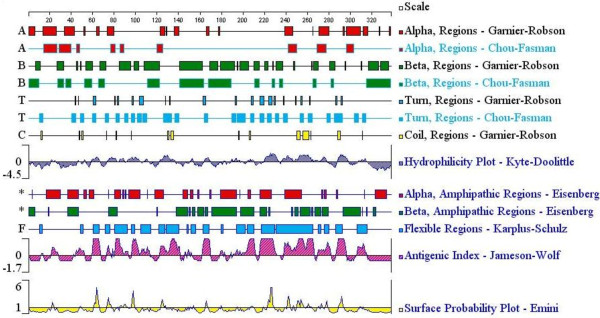
**Secondary structures, flexibility, hydrophilicity, surface probability and antigenicity index for*T. gongdii *SAG1*.***

**Figure 3:**
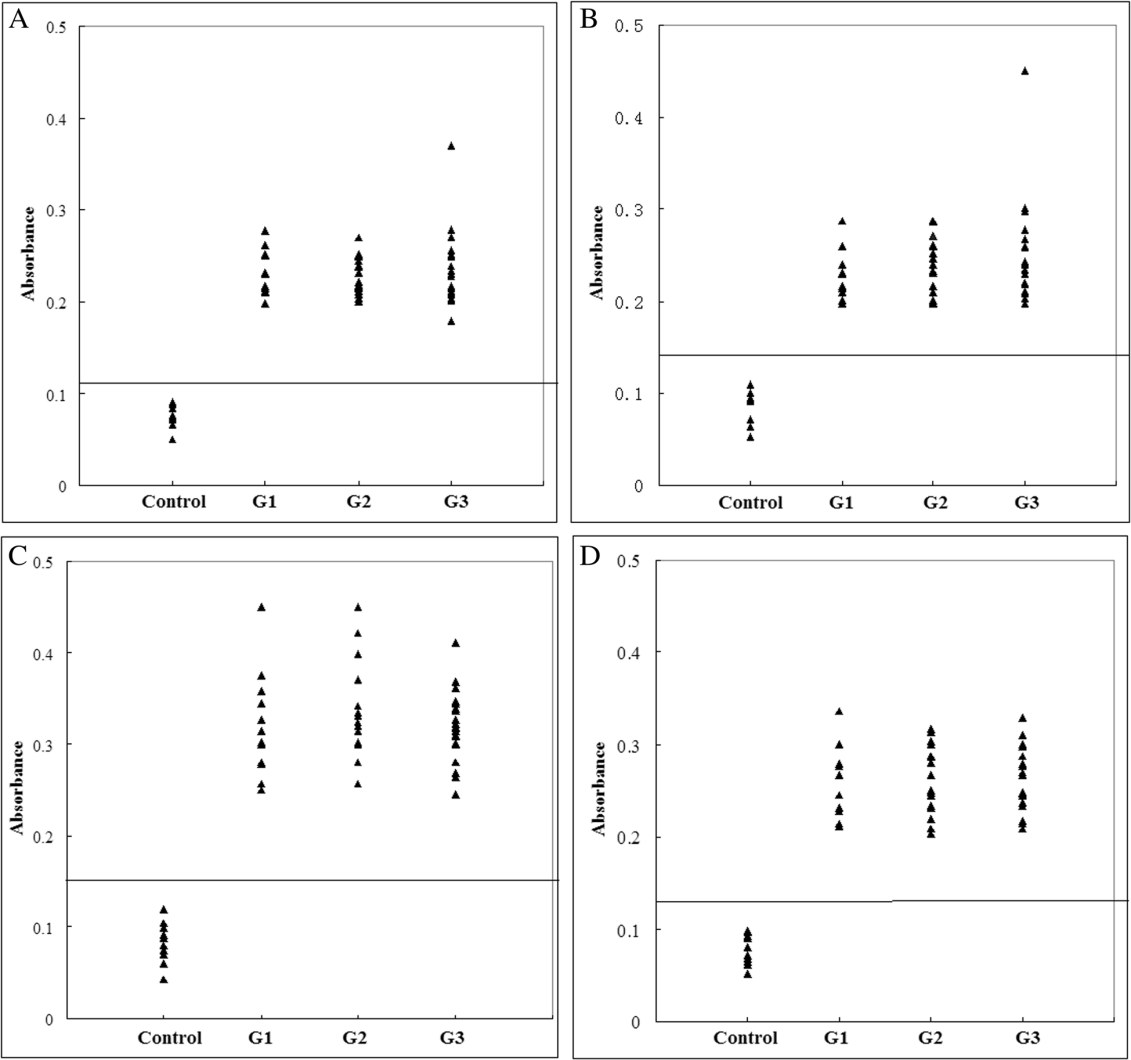
**ELISA of IgG antibodies against different peptides in four groups of pig sera.** (**A**), (**B**), (**C**) and (**D**) show the absorbances targeting to PS4-2, PS6-3, PS10-3 and PS11-2, respectively. The cut-off point for the assay is indicated by the horizontal line.

To further determine the epitopes of SAG1 and decrease the number of laboratory experiments, bioinformatics was used to predict the epitopes. The secondary structure and the surface properties of the SAG1 were analyzed as described by Zhang [12]. The results are shown in Figure 2. Based on these results, 9 shorter peptides that were derived from PS4, PS6, PS10 and PS11 were chosen for further investigation (Table 1). These peptides were tested using pig sera as described above. Four out of 9 peptides (PS4-2, PS6-3, PS10-3 and PS11-2) were recognized by all sera. The results are shown in Figure 3.

## Conclusion

Conformational epitope selection needs to determine the tertiary structure of antigen in order to identify antibody interacting residues in antigen. Experimental techniques such as crystallography are expensive and time consuming. Thus, our work focuses on linear epitope selection. In this study, we have located the epitopes of *T. gondii* SAG1 to a shorter sequence than had been identified previously. The identified epitopes will be useful in vaccine and diagnostic reagent design.

## Competing interests

The authors declare that they have no competing interests.

## Authors’ contributions

YHW, HY and DLZ designed the experiment. YHW, GXW and MW performed lab work and drafted the manuscript. All the authors read and approved the final manuscript.
